# Treatment of patients with Graves’ disease in Sweden compared to international surveys of an ‘index patient’

**DOI:** 10.1002/edm2.244

**Published:** 2021-03-16

**Authors:** Gabriel Sjölin, Kristina Byström, Mats Holmberg, Ove Törring, Selwan Khamisi, Jan Calissendorff, Mikael Lantz, Bengt Hallengren, Helena Filipsson Nyström, Tereza Planck, Göran Wallin

**Affiliations:** ^1^ Faculty of Medicine and Health Örebro University Hospital Örebro Sweden; ^2^ Department of Medicine Örebro University and University Hospital Örebro Sweden; ^3^ Institute of Medicine Sahlgrenska Academy University of Gothenburg Göteborg Sweden; ^4^ ANOVA, Karolinska University Hospital Stockholm Sweden; ^5^ Institution for Clinical Science and Education Karolinska Institutet Stockholm Sweden; ^6^ Department of Endocrinology Uppsala University Hospital Uppsala Sweden; ^7^ Department of Medical Sciences Uppsala University Uppsala Sweden; ^8^ Department of Molecular Medicine and Surgery Karolinska Institutet Stockholm Sweden; ^9^ Department of Endocrinology, Metabolism and Diabetes Karolinska University Hospital Stockholm Sweden; ^10^ Department of Endocrinology Skåne University Hospital Malmö Sweden; ^11^ Department of Clinical Sciences Lund University Lund Sweden; ^12^ Department of Endocrinology Sahlgrenska University Hospital Göteborg Sweden; ^13^ Wallenberg Center for Molecular and Translational Medicine Göteborg Sweden

**Keywords:** antithyroid drugs, Graves’ disease, hyperthyroidism, index patient, international surveys, long‐term follow‐up, radioactive iodine, thyroidectomy, treatment options

## Abstract

**Introduction:**

The treatment strategies for a 42‐year‐old female index patient with moderate Graves’ disease (GD) vary according to several international surveys. The important question whether surveys of treatment preferences in theoretical patient cases also match how real patients are treated has not yet been addressed.

**Materials and Methods:**

From a Swedish cohort of 1186 GD patients (TT‐12 cohort), 27 women were identified using the same criteria as from the index patient surveys from the European and American Thyroid Associations. This ‘index patient cohort’ was age 40–45, otherwise healthy female, with two children and uncomplicated GD. The applied first‐line treatment of the patients in the index cohort, together with its variations, was compared with the treatment preferences according to international surveys. A comparison with the TT‐12 cohort was also performed.

**Results:**

In the ‘Index cohort’, 77.8% were treated with antithyroid drugs (ATD), and 22.2% were treated with radioiodine (^131^I). This preference for ATD is in line with most countries/regions, with the exception of USA and the Middle East/North Africa, where ^131^I was preferred. The distribution of treatment in the TT‐12 cohort did not significantly differ from the index cohort. ATD was the preferred treatment in male and young (age 19–22) patients, as was RAI in old (age 69–73) patients. The age‐related, but not the gender‐related, cases differed significantly from the entire TT‐12 cohort.

**Conclusion:**

The treatment choice in an index patient in Sweden seems in line with European practice, where ATD is the preferred first choice. This differs compared to US and North African survey intentions, where ^131^I is more often used. Age more than gender influences the treatment choice of GD patients. This is, to our best knowledge, the first time an index patient from ‘real life’ has been presented and compared to treatment preferences of international thyroid association surveys.

## Graphical abstract

According to several international surveys, the treatment strategies for a 42‐year‐old female index patient with moderate Graves’ disease vary considerably. In this study, the correlation of treatment strategies between patients from the Swedish thyrotoxicosis 2012 study cohort, who met the criteria for the index patient and its variations, and the theoretical index patient in international surveys is examined and compared. This is in order to allow a reasonable comparison with and, if possible, to validate the international surveys.

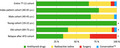

## 1 INTRODUCTION

Graves’ disease (GD) is a common autoimmune disease with an incidence of 6–93 per 100,000 population.^1^, ^2^, ^3^ It has three main treatment options: antithyroid drugs (ATD), radioactive iodine (^131^I) or surgery. GD mostly affects women at 30–60 years of age.^2^

The treatment preferences of a hypothetical ‘index patient’ vary internationally, as illustrated by several authors.^4^, ^5^, ^6^, ^7^, ^8^, ^9^ Several factors influence the choice of treatment, such as age, sex, the patient's preferences, planned pregnancy, smoking, eye symptoms and cost‐effectiveness^10^, ^11^; additionally, local traditions and access to treatments can influence the choice of treatment. These issues may explain why different treatment strategies are used for an ‘index case’ of GD.^4^, ^5^, ^6^, ^7^, ^8^, ^9^

Several clinical studies showed that ATD is a common first choice of treatment of GD worldwide, and treatment strategies imply a higher use of ATD in South East Asia and in Europe than in the USA, where ^131^I is predominantly used.^12^, ^13^, ^14^, ^15^, ^16^

Surgery, on the other side, is less frequently used as a first‐line treatment option, but it is more common in France (6.1%) and Sweden (4.6%) than in the USA, Taiwan and South Korea (2.0–2.9%).^12^, ^13^, ^14^, ^15^, ^16^

These clinical studies do not take into account the previously mentioned factors influencing the treatment choice. To accommodate this problem, the European Thyroid Association (ETA), Japan Thyroid Association (JTA) and American Thyroid Association (ATA) conducted index patient questionnaire (IPQ) surveys, asking the responding physicians how they would treat a hypothetical index patient under various circumstances (Table 1). At least 14 Thyroid Association surveys of treatment preferences for an index patient from over 10 countries or regions, spanning more than three decades, have been published.^4^, ^5^, ^6^, ^7^, ^8^, ^9^, ^25^ The documentation illustrates that the preferences for treatment not only depends on the country or region but also differs in treatment strategies chosen during various time‐periods.

**TABLE 1:** Original criteria for the Index patient

Original criteria for the index patient	Data in our database	Exception
43‐year‐old active otherwise healthy woman	Yes	
Overt signs and symptoms of 2–3 months of duration	No	
First episode	Yes	
On no medications	Yes	
Has two children ages 5 and 10 with no plans for more	Yes	Age of children not included
Thyroid diffusely enlarged at 40–50 g	Yes	All patients presumed to have a goitre less than 40 g in size^a^
Minimal eye signs	No	
Pulse 105/min	No	

Glinoer et al.^35^

^a^ Hallgrimson.^26^

The important question, whether a survey of treatment preferences based on theoretical patient cases also matches how real patients, similar to an ‘index patient’, is treated today, has not been elucidated. In this study, we therefore present the actual treatment choices in Sweden for patients who meet the criteria for an index patient used in international surveys.

We aimed to study the following:

1. The choice of treatment of consecutively registered patients, who fulfilled the criteria for the index patient and its variations, in a Swedish national cohort.
2. How the treatment for the Swedish ‘clinical index cohort’ compares to the rest of the Swedish national cohort (TT‐12 cohort) and the international surveys of treatment preferences for the theoretical index patient.
3. Do index patient questionnaire surveys represent the real actual clinically treatment situation?

## 2 MATERIAL AND METHODS

### 2.1 Study design

Otherwise healthy patients with uncomplicated GD, age 40–45, were selected from a previously established cohort of Swedish GD patients.^16^ From this cohort, the thyrotoxicosis 2012 study (TT‐12), the index patients were recruited. Comparisons were made with several other index patient questionnaires (IPQ) surveys from different international thyroid associations around the world.^4^, ^5^, ^6^, ^7^, ^8^, ^9^, ^25^ Cohorts of index male patients, young patients (19–22 years old), older patients (69–73 years old) and patients with a relapse after ATD were also examined as variants of the index case.

### 2.2 Subjects

The patients were collected from an original Swedish cohort (the TT‐12 cohort) consisting of 1186 adult GD patients followed up 6–10 years after diagnosis from 2003–2005.^16^ The initial cohort was collected over three years from hospitals covering approximately 40% of the Swedish population. With a loss of approximately 46% to follow‐up, this cohort corresponds to approximately 65% (40% of population x 3 years x 54%) of the total yearly incidence of GD in Sweden.

### 2.3 Definition of the index patient

Patients as similar as possible to the index case of a 42‐ to 43‐year‐old otherwise healthy woman, with two children, and uncomplicated GD, were extracted from the database with the same criteria used as in the previous ETA and ATA surveys^4^, ^5^, ^6^, ^7^ (Table 1). The definitions of the index patient have, in small ways, changed over time and between the different surveys. For example, the age of the index patient changed from the first study to more recent studies, from 43‐year‐old to 42‐year‐old patient. Where such differences occur, the original definition from the first ETA survey^4^ has been used. Taken together, there are 10 variations of the index case, and we collected data for four sub‐cohorts of actual patients fulfilling these criteria.

Since most patients in Sweden have GD without a large goitre, we presume all selected patients had a goitre size less than 40–50 g. The mean thyroid weight in GD of cases undergoing surgery in Sweden is 30 g.^26^

There were no records of symptoms or the age of the woman's two children, and therefore, these criteria had to be excluded. Additionally, when selecting the patients, only five 42‐year‐old and three 43‐year‐old female patients were found. The age criterion was extended to include all female patients of close ages, that is, 40–45 years old, which resulted in 27 patients. This adjustment is considered appropriate, because the age range in the surveys was arbitrarily selected, and with a small variation in age definition, the survey responses would probably not change significantly.

Selected patient groups: male, young, old and patients with a relapse.

We also selected young, old and male patients and patients with relapse for separate analyses.

TT‐12 male cohort: There were only two 43‐year‐old males in the database. Using the extended age group of 40–45 years old as in the index case leads to a group of 31 male patients.

TT‐12 young female cohort: In the earlier surveys, young patients were defined as a 19‐year‐old woman and in the later investigations as a 22‐year‐old woman. Since there were only six 19‐year‐old and four 22‐year‐old women in our database, the criterion was set to an age 19–22, resulting in 13 included patients. There was also, in the later surveys, a case with a woman who wished to get pregnant. Since there were no questions about pregnancy wishes in our questionnaire, these patients were assumed to be equivalent to the young patients, supposing all young women may have a wish for pregnancy.

TT‐12 old female cohort: The old index patient in previous investigations was defined as a 71‐year‐old woman, but since there were only five patients fulfilling this criterion in our study, we expanded the group to an age 69–73, resulting in 33 patients.

TT‐12 relapse female cohort: Concerning the cases with relapse after ATD and surgery, we can only report on the relapse after ATD since there were nine female patients between 40–45 years old who later had a relapse after primary ATD treatment in this cohort, but none after surgery.

### 2.4 Statistical analysis

To calculate significant differences between the different groups, which consist of different types of data, 95% confidence intervals (CIs) of proportions were calculated for our index groups. If, then, the entire original cohort or any of the results from the IPQ surveys falls outside that range, they are considered to have a significant difference.

The IPQ surveys all have a low proportion of surgically and conservatively (symptomatic treatment with beta‐blockers) treated patients, and this is also true for the whole TT‐12 cohort, so the only real difference is in the ratio between ATD and ^131^I.^4^, ^5^, ^6^, ^7^, ^8^, ^9^, ^25^ In this study, we report the rate of ATD treatment in Sweden compared to suggested rates in the rest of the world. To use the same methodology comparing other clinical studies with relevant IPQ surveys, confidence intervals were calculated from their results.

### 2.5 Ethics

This study was approved, as a part of the TT12 project, by the Regional Ethics Committee in Uppsala (Dnr 2012/035, 2012 April 4). The study was performed according to the Declaration of Helsinki.

## 3 RESULTS

### 3.1 Index patient (40–45 years old)

**FIGURE 1:**
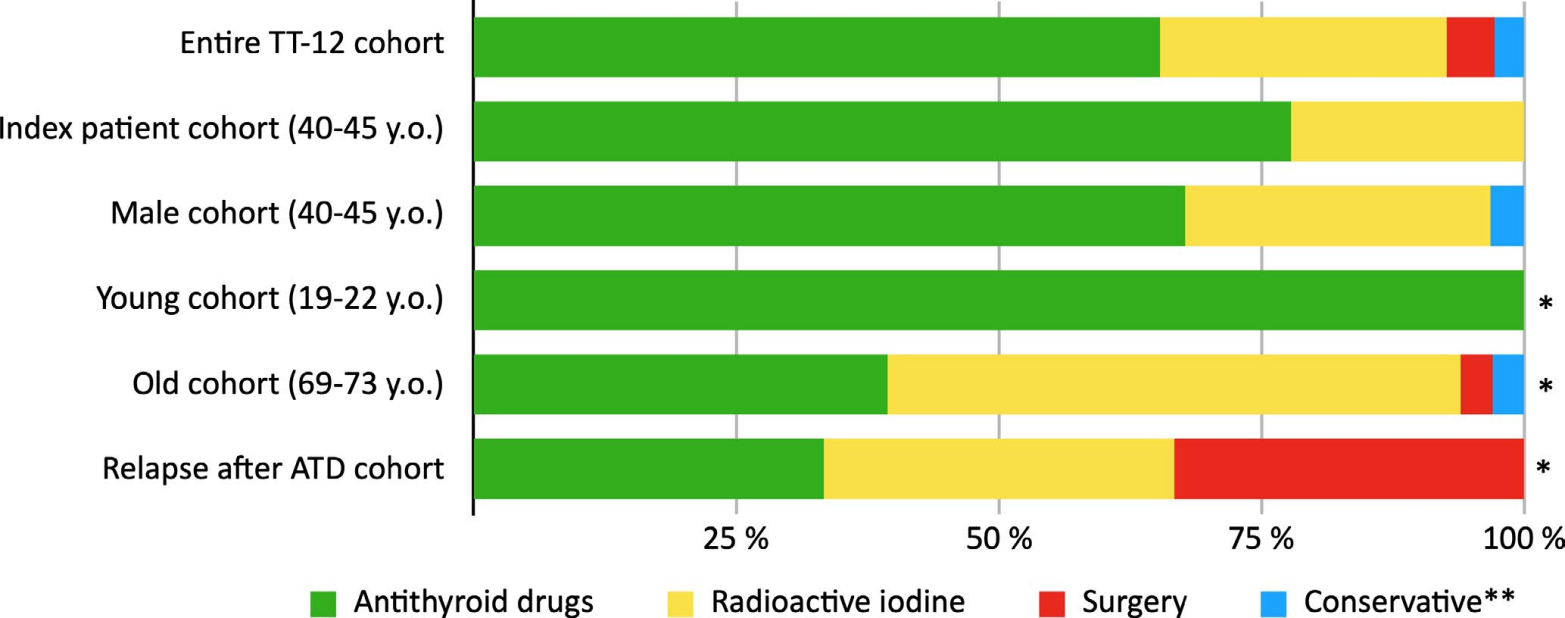
Treatment of an index patient, with, variations, in the TT‐12 cohort. Comparison between the TT‐12 cohort and an index patient cohort, with variations, concerning the distribution of first‐line treatment with ATD, ^131^I and surgery for Graves’ disease. *The results from the entire TT‐12 cohort are outside the confidence interval of the group. ** Symptomatic treatment with beta‐blockers

**FIGURE 2:**
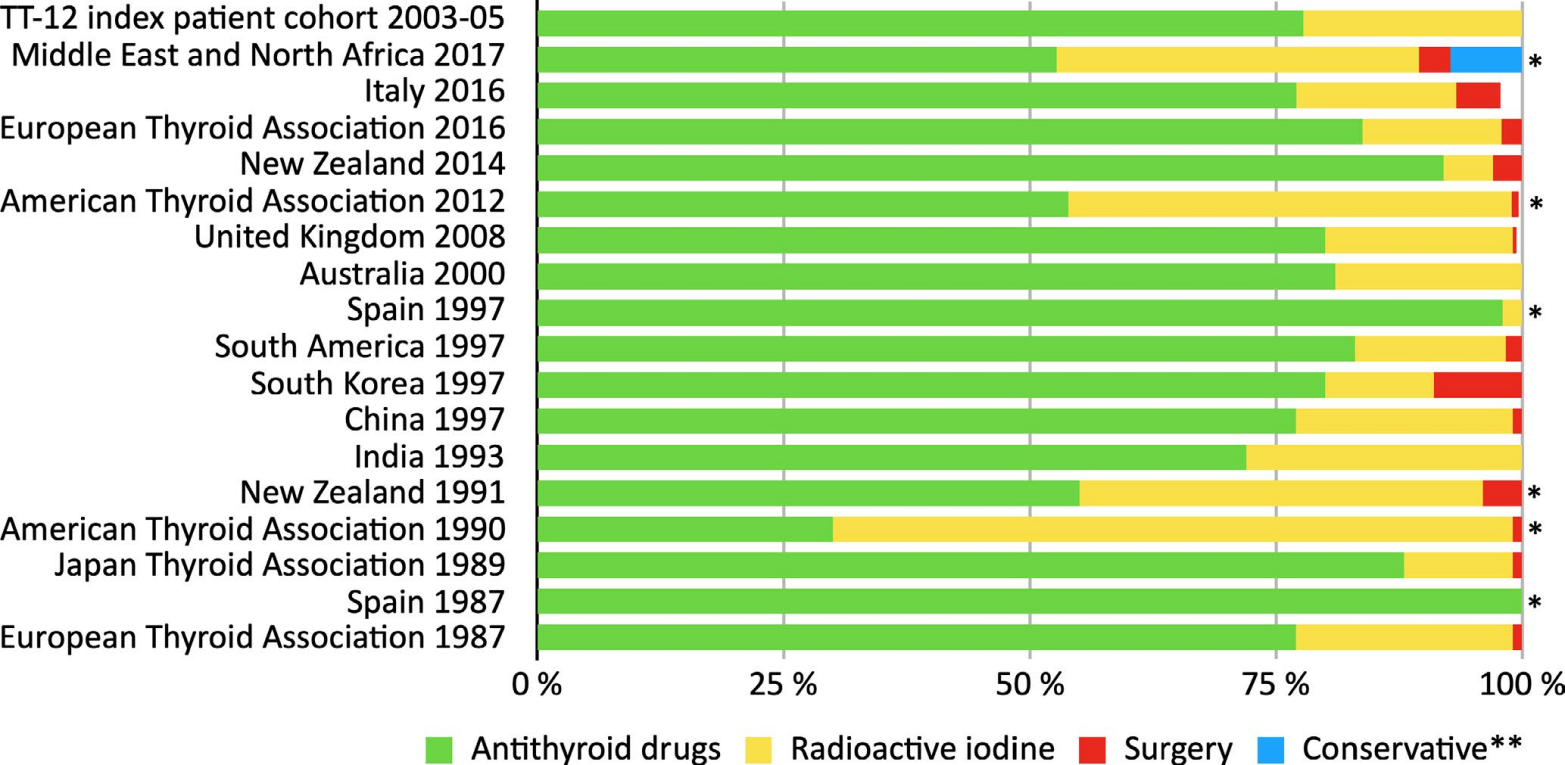
Comparison between our cohort and TA studies concerning the treatment of the index patient. Distribution of first‐line treatment of an index patient with ATD, ^131^I and surgery for Graves’ disease in different countries over the past 30 years. *The results are outside the confidence interval of our index cohort. ** Symptomatic treatment with beta‐blockers. Missing data in material from Italy 2016 and UK 2008 represented with white

Of the 27 female patients meeting the criteria for the index case, 77.8% (CI 62.1–93.5%) received ATD, while 22.2% were given ^131^I. There was no significant difference compared to the entire TT‐12 cohort, of which 65.3% received ATD and 27.3% ^131^I treatment (shown in Figure 1). A comparison between the TT‐12 index patient cohort and the IPQ surveys can be seen in Figure 2. ATD was used at a similar frequency compared to the TT‐12 index patient cohort in most countries/regions, with the exception of the USA and Middle East/ North Africa, where ^131^I was preferred.

### 3.2 Special patient groups in the Swedish cohort

ATD treatment was the most common (67.7% CI 51.2–84.2%) treatment in the 31 male patients aged 40–45 years, with ^131^I as the second most common treatment (29.0%). No male patient received surgical treatment, but 3.2% of the patients were treated conservatively with beta‐blockers. This male group did not significantly differ from the entire TT‐12 cohort (shown in Figure 1). This is not significantly different from most IPQ surveys, with the exception of the USA and Spain.

**FIGURE 3:**
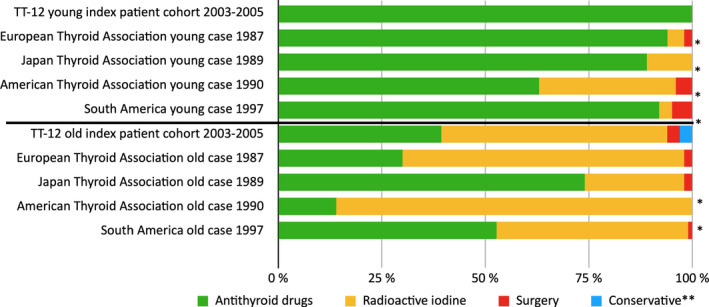
Comparison between our cohort and TA studies concerning the treatment of the Young and Old variation of the index patient. Distribution of first‐line treatment of Young (19–22 years old) and Old (69–73 years old) index patients with ATD, ^131^I and surgery in Graves’ disease in different countries. *The results are outside the confidence interval of our index cohort. ** Symptomatic treatment with beta‐blockers

All 13 patients aged 19–22 years were treated with ATD (100% CI 100–100%). This differs significantly from the entire TT‐12 cohort (shown in Figure 1) and all IPQ surveys, except Spain (shown in Figure 3).

Concerning the 33 older (69–73 years old) patients, there was a significant difference in relation to the entire TT‐12 cohort, since the ratio between ATD and ^131^I was almost inverted compared to the index case. ^131^I was used in 54.5% of the cases and ATD only in 39.4% (CI 22.7–56.1%). Additionally, surgery and conservative treatment with beta‐blockers were used as viable options, with a usage of 3.0% each (shown in Figure 1). The TT‐12 old female patient adhered to the preferences of Europe and South America, but not to surveys from Japan or the USA (shown in Figure 3).

In the nine patients who had a relapse after ATD treatment, the secondary treatments were equally divided (33.3% CI 2.5–64.1%) between ATD, ^131^I and surgery. This distribution was significantly different from the entire TT‐12 cohort (shown in Figure 1). This is not significantly different compared to most IPQ surveys, with the exception of the USA.

### 3.3 Other clinical studies

The calculated CI for the South Korean clinical investigation^13^, where 97.4% (CI 95.1–99.7%) received ATD, is significantly different from the suggested 80% in the South Korean IPQ survey.^27^ Only 16.4% (CI 13.7–19.1%) received ATD in an American clinical study,^14^ compared to 30% and 53.9% in the ATA surveys.^6^, ^8^ In Europe, the ETA surveys showed 77% and 83.8% ATD preferences.^4^, ^9^ This differs significantly from the French study,^12^ with 91% (CI 89.2–92.8%) ATD usage and even from the whole Swedish TT‐12 cohort with 65.3% (CI 62.6–68.0%) ATD usage.^16^

## 4 DISCUSSION

Most clinical studies do not match the findings of the corresponding national/regional IPQ survey, which casts doubt on the validity of these studies. By only asking for the physician's view, the IPQ surveys exclude the patient's own choice. The patient's choice of treatment is important and in Sweden should always be considered according to the Swedish Health and Medical Services Act. Therefore, the IPQ surveys stand in contrast with clinical studies, where not only the physicians’ recommendations are considered, but the selection is much wider than for an index case, making the IPQ surveys a weak substitute for clinical studies. Even though IPQ surveys inappropriately approximate reality, they still might be able to measure trends over time and between countries/regions.

The treatment choices of the TT‐12 index patient cohort, together with its variations, all adhere to the results of the ETA surveys, with the exception of the young age group and differ significantly from the ATA surveys in which treatment with ^131^I was more common in both young and old patients.^4^, ^6^, ^8^, ^9^ These differences could in some meaning be attributed to differences in the ATA and ETA guidelines,^11^, ^28^ as the Swedish doctors seem to adhere to the latter.

A comparison between a real patient cohort and IPQ surveys ^4^, ^5^, ^6^, ^7^, ^8^, ^9^, ^25^ is relevant, even if the gender proportion in the patient group examined differs from the gender distribution normally seen in GD. This is illustrated by the observation that there is no difference with regard to the treatments provided between the entire TT‐12 cohort and both the female and male TT‐12 index patient cohorts. This is most likely because of the reluctance to use ^131^I in women of childbearing age. So, age more than gender influences the treatment choice of GD patients. Thus, a comparison of a clinical cohort and IPQ surveys should be made carefully, since the composition of the cohort may differ from the criteria of the index patient. This can be seen in a comparison of clinical studies and the relevant IPQ surveys.^4^, ^6^, ^8^, ^9^, ^12^, ^13^, ^14^, ^16^, ^27^ In the end, there is a difference between studying constructed cases and treated populations; ‘fiction’ does differ from reality. IPQ surveys may reflect the treatment intention of the medical community, but to study what treatment patients receive is the only way is to conduct clinical studies. The differences between countries, as illustrated in the IPQ surveys, are possibly due to different circumstances in both the community and the health‐financial system. It may, to some extent, also illustrate a difference in the perception of cure between different countries/regions.

Antithyroid drugs is the primary treatment preference of a 42‐year‐old female index patient with moderate GD (Table 1) in most of the world, according to IPQ surveys, as it was with the TT‐12 index patient cohort. The exceptions are the USA, Middle East/ North Africa, where ^131^I is preferred.^4^, ^5^, ^6^, ^7^, ^8^, ^9^, ^25^ This clearly shows the differences that exist in treatment strategies. Perhaps it also highlights that there may not only be a difference in the choice of treatment, but perhaps also in the view of treatment and cure. If only the reversal of hyperthyroidism is considered, ablative treatments are the best choice, even though the risk of substitution with levothyroxine is higher. On the other hand, if cure is defined as ‘free from pharmaceuticals’ ^131^I might be a less good choice, as the payoff between the risk of recurrence and risk of substitution treatment maybe better with the non‐ablative ATD treatment.^16^

In regions with repeated IPQ surveys, ETA (1987, 2016), ATA (1990, 2012) and New Zealand (1991, 2014), a trend of using more ATD can be seen.^4^, ^6^, ^8^, ^9^, ^17^, ^29^ Studies showing that RAI can increase the risk of Graves’ orbitopathy may be one explanation for this trend away from RAI.^30^, ^31^, ^32^, ^33^ Another pattern may be a growing popularity in the use of long‐term ATD therapy.^11^, ^28^ The same pattern seems to hold when looking at a 42‐year‐old male, where most countries/regions prefer ATD before ^131^I.^4^, ^5^, ^18^, ^19^, ^21^ This is also in line with the results of the TT‐12 male cohort. The US centres predominantly uses ^131^I for the same patients.^6^ Perhaps it is a step towards a more internationally aligned treatment strategy.

Antithyroid drugs is the main choice for the TT‐12 young (19–22 years old) female patient, as it is in most countries/regions,^4^, ^5^, ^6^, ^24^, ^25^ but an interesting trend in these patients is that surgery has gained popularity in some of these cases, for example, Europe and the USA show a 20–30% operation preference of patients in this age group.^8^, ^9^ The change over time could be attributed to the difference in the variations of the index cases introduced over time. In the later studies, both the young age and the desire for pregnancy are taken into account, as opposed to only the young age in the previous studies. Since, clinically, one cannot ignore a potential wish for pregnancy in young women, this point is probably moot. This also sheds light on another flaw in the index patient, as women today may have a reasonable wish for pregnancy up to 35–45 years of age.^34^

When comparing the treatment of older patients (69–73 years old), most countries, including the TT‐12 old female cohort, prefer ^131^I rather than ATD, and only Spain (in 1997, but not in 1987) and Japan use ATD as the primary treatment.^4^, ^5^, ^6^, ^18^, ^19^, ^20^, ^21^, ^22^ Second‐line treatment after relapse after ATD treatment is predominately ^131^I in Europe, USA and South America, which is not significantly different from the TT‐12 relapse female cohort. However, in contrast, a second ATD treatment is more common in Japan.^4^, ^5^, ^6^, ^18^, ^19^, ^21^, ^22^ This is a striking difference. Japan has a history of aversion to radioactivity, given the two atomic bombs ^20^ and, most recently, a nuclear accident.

Since both the old and relapse case is not reiterated in later surveys, these results are old (before year 2000) and much has happened to treatment strategies since then. Therefore, the results from these IPQs are not up to date, but the comparison with patients from 2003 to 2005 is still relevant and illustrates that these variations are relevant since they adhere to the relevant TT‐12 cohorts, but not the whole TT‐12 cohort. Further on, this study shows how difficult it is to compare IPQ studies with population studies. Comparisons between a clinical study and an IPQ survey are only reliable when the cohort is selected with the same criteria as the index case. This is based on the premise that the young and old patient groups differ significantly from the entire TT‐12 cohort, as both the Female and the Male TT‐12 cohorts do not.

This can also be seen when looking at other clinical studies. One of the most interesting findings is South Korea, where both an IPQ survey and a clinical study have been conducted.^13^, ^20^ The South Korean IPQ survey shows a 20% significantly lower preference for ATD compared to what the patients received in the correlating clinical study. This could depend on the differences in the composition of the cohort and the index criteria, like the fact that 25% of the South Korean cohort has TAO.^13^

The same discrepancy between clinical studies and IPQ surveys can also be seen in America where only 16% of patients received ATD in an American clinical study^14^ compared to 30% and 54% in the early and late ATA surveys.^6^, ^8^ These studies, like many population studies, are, however, single centre studies with a relatively more homogenous treatment practice than in the IPQ surveys. Nevertheless, the same pattern can still be seen when comparing IPQ surveys with national multicentre studies in Europe. There are differences in the preference for ATD between the ETA surveys^4^, ^9^ (77% to 83.8% preference for ATD) in comparison with national multicentre studies both in French (91% preference for ATD)^12^ and Sweden (65% preference for ATD).^16^

Index patient questionnaire surveys span, not only over countries, but sometimes continents, making the comparisons even with nationwide studies difficult. A student report, in form of a IPQ survey, from Sweden in 2005 (not published) shows regional differences in treatment strategies, with a range of ATD preference in an index patient from about 15%–100%. This is also corroborated in the precursor of the TT‐12 cohort where regional differences in treatment can be seen,^1^ making even the comparison of national IPQ surveys and populations studies hard. At the end, since the validity of the IPQ surveys is in doubt, a proper validation of the IPQ surveys should be done.

The small size of the selected groups provides a wide confidence interval, which makes the results more uncertain. Nevertheless, differences between the groups, the IPQ surveys and the entire TT‐12 cohort, can still be seen. The database also did not contain all of the variables for the selection, so the groups may not be quite the same as the index cases. Another limitation is that treatment strategies have changed since 2003–2005, when patients received their first treatment in this study. They are, however, compared with physicians’ treatment preferences from a 25‐year perspective, where these years are in the centre. This is both a strength and a limitation. No survey is conducted exactly at the same time as this study, which complicates comparisons. However, most surveys were conducted less than 10 years before or after this study, which means that comparisons could be viewed as a type of mean.

The main strength of this investigation is the large database that makes it possible to select patients according to these criteria and obtain analysable groups. Even though the index patient has been described many times in the literature, this is, to our best knowledge, the first time an index patient from ‘real life’ has been presented and compared to the treatment preferences of IPQ surveys.

## 5 STATEMENT OF ETHICS

The study was approved, as a part of the TT12 project, by the Regional Ethics Committee in Uppsala (Dnr 2012/035, 2012 April 4). The study was performed according to the Declaration of Helsinki.

## CONFLICT OF INTEREST

Gabriel Sjölin, Kristina Byström, Mats Holmberg, Ove Törring, Selwan Khamisi, Jan Calissendorff, Mikael Lantz, Bengt Hallengren, Helena Filipsson Nyström, Tereza Planck, Göran Wallin: none of the authors have competing financial interests.

## AUTHOR CONTRIBUTIONS

Gabriel Sjölin has made a substantial contribution to the design, acquisition, analysis and interpretation of data, as well as drafting the work. Kristina Byström has made a substantial contribution to the acquisition and interpretation of data, as well as revising it critically for important intellectual content. Mats Holmberg has made a substantial contribution to the acquisition and interpretation of data, as well as revising it critically for important intellectual content. Ove Törring has made a substantial contribution to the conception and design of the project, acquisition and interpretation of data, as well as revising it critically for important intellectual content. Selwan Khamisi has made a substantial contribution to the acquisition and interpretation of data, as well as revising it critically for important intellectual content. Jan Calissendorff has made a substantial contribution to the acquisition and interpretation of data, as well as revising it critically for important intellectual content. Mikael Lantz has made a substantial contribution to the acquisition and interpretation of data, as well as revising it critically for important intellectual content. Bengt Hallengren has made a substantial contribution to the acquisition and interpretation of data, as well as revising it critically for important intellectual content. Helena Filipsson Nyström has made a substantial contribution to the acquisition and interpretation of data, as well as revising it critically for important intellectual content. Tereza Planck has made a substantial contribution to the interpretation of data, as well as revising it critically for important intellectual content. Göran Wallin has made a substantial contribution to the design, acquisition and interpretation of data, as well as revising it critically for important intellectual content. All approve the final version to be published and agree to be accountable for all aspects of the work in ensuring that questions related to the accuracy or integrity of any part of the work are appropriately investigated and resolved.

## Data Availability

Data available on request due to privacy/ethical restrictions.
